# Free-living core body temperature monitoring using a wrist-worn sensor after COVID-19 booster vaccination: a pilot study

**DOI:** 10.1186/s12938-023-01081-3

**Published:** 2023-03-13

**Authors:** Samuel Etienne, Ruben Oliveras, Giovanni Schiboni, Lukas Durrer, Fabien Rochat, Philipp Eib, Michele Zahner, Michael Osthoff, Stefano Bassetti, Jens Eckstein

**Affiliations:** 1 Division of Internal Medicine, University Hospital Basel, Petersgraben 4, 4031 Basel, Switzerland; 2 greenTEG AG, Zurich, Switzerland; 3 Department of Clinical Research, University of Basel, Basel, Switzerland; 4 Department Digitalization and ICT, University Hospital Basel, Basel, Switzerland

**Keywords:** Remote monitoring, Wearable monitoring, Wearable sensor, Wrist-worn sensor, Free-living, Core body temperature, Fever, SARS-CoV-2, COVID-19 pandemic, Telemedicine

## Abstract

Core body temperature (CBT) is a key vital sign and fever is an important indicator of disease. In the past decade, there has been growing interest for vital sign monitoring technology that may be embedded in wearable devices, and the COVID-19 pandemic has highlighted the need for remote patient monitoring systems. While wrist-worn sensors allow continuous assessment of heart rate and oxygen saturation, reliable measurement of CBT at the wrist remains challenging. In this study, CBT was measured continuously in a free-living setting using a novel technology worn at the wrist and compared to reference core body temperature measurements, i.e., CBT values acquired with an ingestible temperature-sensing pill. Fifty individuals who received the COVID-19 booster vaccination were included. The datasets of 33 individuals were used to develop the CBT prediction algorithm, and the algorithm was then validated on the datasets of 17 participants. Mean observation time was 26.4 h and CBT > 38.0 °C occurred in 66% of the participants. CBT predicted by the wrist-worn sensor showed good correlation to the reference CBT (*r* = 0.72). Bland–Altman statistics showed an average bias of 0.11 °C of CBT predicted by the wrist-worn device compared to reference CBT, and limits of agreement were − 0.67 to + 0.93 °C, which is comparable to the bias and limits of agreement of commonly used tympanic membrane thermometers. The small size of the components needed for this technology would allow its integration into a variety of wearable monitoring systems assessing other vital signs and at the same time allowing maximal freedom of movement to the user.

## Introduction

Core body temperature (CBT) is an important vital sign (VS) and fever is an important indicator of disease [1]. In humans, CBT is closely controlled around its normal value of 36.5 °C with 24-h variations of only 1.5 °C. Fever represents a controlled deviation of the host from the otherwise precisely maintained temperature homeostasis and is a complex adaptive response to several immune challenges whether infectious or non-infectious [2]. “Fever” is also the single most frequently reported manifestation of coronavirus disease 2019 (COVID-19) and has been reported to be one of the earliest signs of symptomatic COVID-19 [3, 4]. Therefore, CBT monitoring plays an important role both in the surveillance of infected individuals and in screening programs.

The gold standard for the determination of CBT is pulmonary artery catheter measurement, but this method is invasive and time-consuming. Other techniques to determine CBT are measurement of rectal, bladder, and oesophageal temperature, which are also invasive and not practicable in many settings. Ingestible telemetric temperature sensors provide CBT measurements in good agreement with oesophageal or rectal thermometers and are often used for outpatient field-based studies [5]. However, these telemetric pills may be excreted as soon as 8 h after ingestion, limiting their use for CBT monitoring in clinical settings. Peripheral temperature measurements are less precise and reliable than CBT measurements but easier to assess. Many methods have been developed to approximate CBT as closely as possible, such as tympanic, axillary, and oral thermometers [6, 7]. Although these techniques allow obtaining an estimation of body temperature easily, the need of close contact of the operator to the potentially infectious individual still limits their use in the context of highly transmittable diseases, such as COVID-19. Commercially available dermal thermometers designed for home use are applied to the individual’s skin with the help of adhesive tape and allow remote and continuous monitoring of CBT. However, such devices often lack precision and thus are not considered to be suitable for clinical use [8]. Methods that continuously record CBT using heat flux provide a reliable measurement of CBT (3 M™ Bair Hugger™ temperature monitoring system [9] (formerly called 3 M SpotOn [10, 11]), Drägerwerk™ Tcore™ temperature monitoring system [12, 13], Medisim™ Temple Touch Pro™ [14]). However, in these systems, the sensors are applied on the subjects’ forehead and permanent electrical supply is needed, thus they are not practical for use in patients outside the operating room (OR) or the intensive care unit (ICU). Recently, a novel technology (CALERA®, greenTEG®, Switzerland [15]) was shown to accurately monitor CBT in hospitalized stroke patients while worn on the chest [16].

In the past decade, there has been a growing interest in the development of remote patient monitoring (RPM) systems and sensors of vital functions embedded in wearable devices [17–20]. The COVID-19 pandemic has further raised the interest for affordable and reliable health technology that allows RPM and that may be used for large-scale screening programs [21–25]. While wrist-worn sensors allow continuous assessment of VS such as heart rate and oxygen saturation with good accuracy, reliable measurement of CBT at the wrist remains challenging [12, 26].

The present study was conducted to evaluate CBT monitoring and detection of elevated CBT at the wrist using a novel device based on the CALERA® technology. Elevated CBT is reported to occur in 20–37% of individuals within two days after administration of the second or third dose of an mRNA-based COVID-19 vaccine [27, 28]. Thus, CBT was measured continuously with a wrist-worn sensor and compared to CBT values acquired with an ingestible telemetric pill in healthy volunteers during 24 h after administration of a COVID-19 booster vaccination.

## Results

### Data construction

**Fig. 1:**
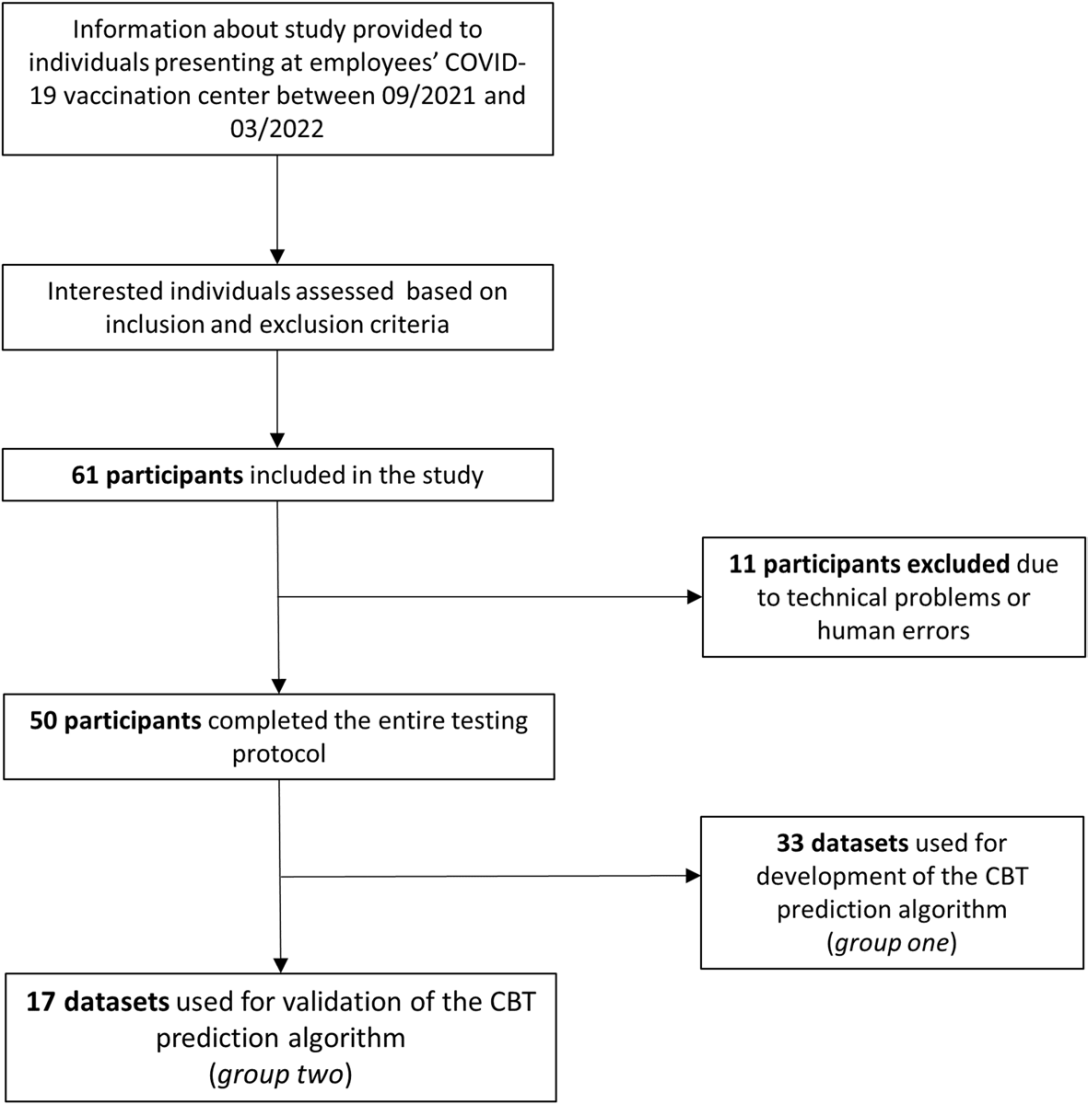
Flow diagram of the study

Sixty-one participants were included in the study. Eleven participants were excluded because of technical issues (e.g., sensor failures) or human errors (e.g., forgetting to turn on the senor) that rendered the measurements invalid. Fifty participants completed the entire study protocol. The dataset was split into two groups with allocation of participants performed to achieve similar distribution of elevated CBT proportion in both groups. The datasets of group one (33 participants) were used for model development and training. The datasets of group two (17 participants) were used for model validation (Fig. 1).

All participants received the mRNA-1273 SARS-CoV-2 vaccine (Moderna™ Spikevax™). The participants reported low physical activity level during the observation time: nearly all participants reported staying at home and resting. Few participants reported low-intensity physical activity, such as household duties (e.g., cleaning or cooking), yoga or outdoor walking. A minority of participants undertook grocery shopping, commuting by bike or home-office working. No adverse event occurred during the study. Important participants’ characteristics are listed in Table 1.

**Table 1:** Participants’ characteristics. The data are reported as mean (standard deviation) or *n* (%)

	Group one (*n* = 33)	Group two (*n* = 17)
Age, years	39.2 (10.0)	35.8 (8.2)
Female participants	19 (57.6)	11 (64.0)
BMI, kg/m^2^	24.1 (4.2)	24.1 (2.8)
Participants receiving second vaccine dose	13 (77)	17 (100)
Participants receiving third vaccine dose	4 (23)	0 (0)
Participants with CBT > 38.0 °C	23 (69.0)	11 (64)
Monitoring duration, hours	26.6 (8.4)	26.1 (9.4)

*BMI* body mass index, *CBT* core body temperature

### Prediction performance

**Fig. 2:**
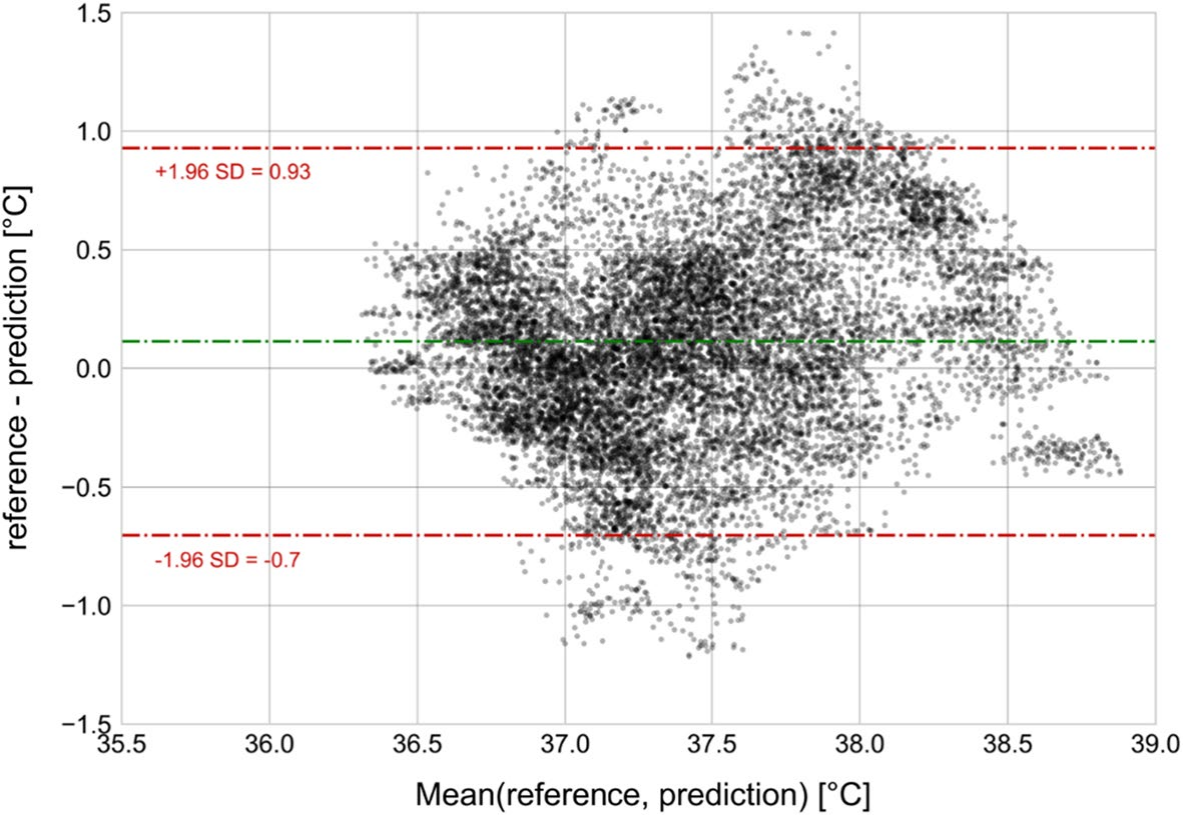
Bland–Altman plot of predicted CBT vs. reference CBT. The green line indicates the bias between the two systems. The red lines indicate 95% limits of agreement

**Fig. 3:**
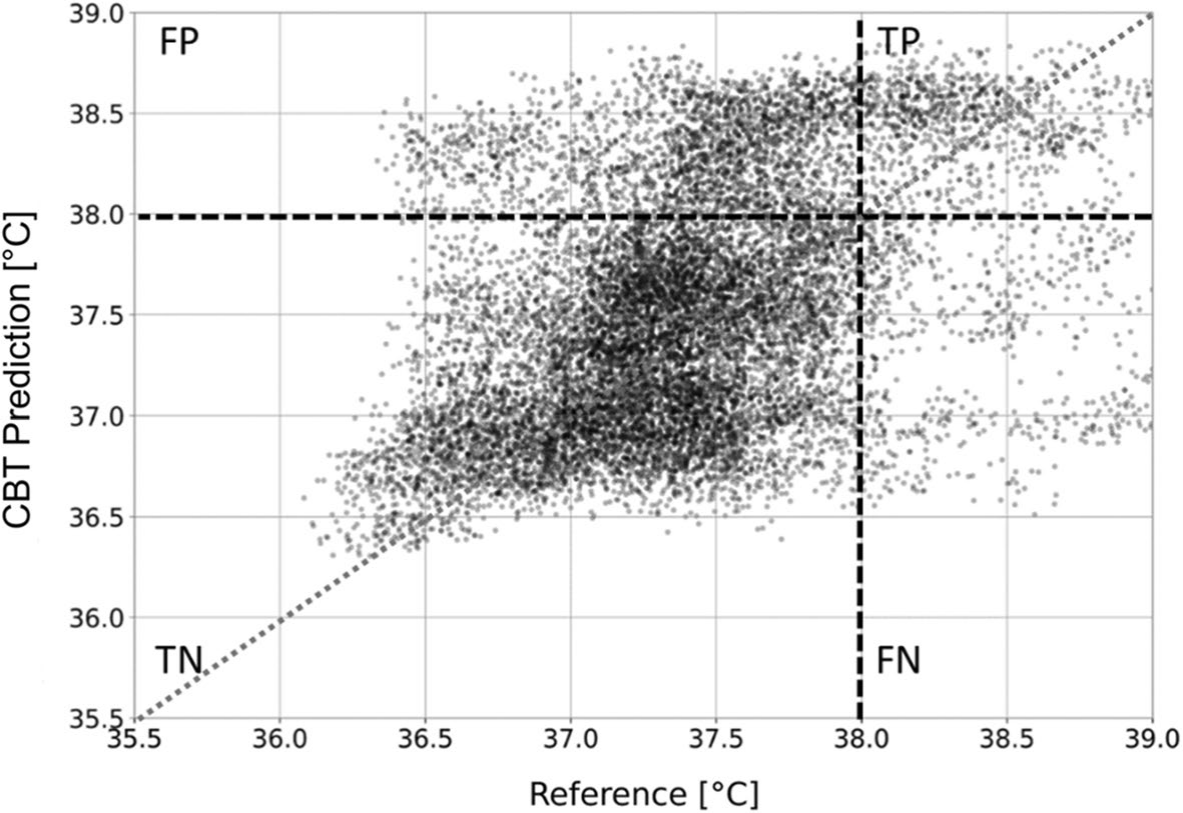
Correlation plot of the predicted CBT and the reference CBT. Predicted CBT is plotted against reference CBT for each measurement. The vertical and horizontal dashed lines indicate the cut-off for “elevated CBT”, defined as > 38.0 °C. The dotted line suggests identity with reference CBT. *CBT* core body temperature, *FP* false positive, *TP* true positive, *TN* true negative, *FN* false negative

**Fig. 4:**
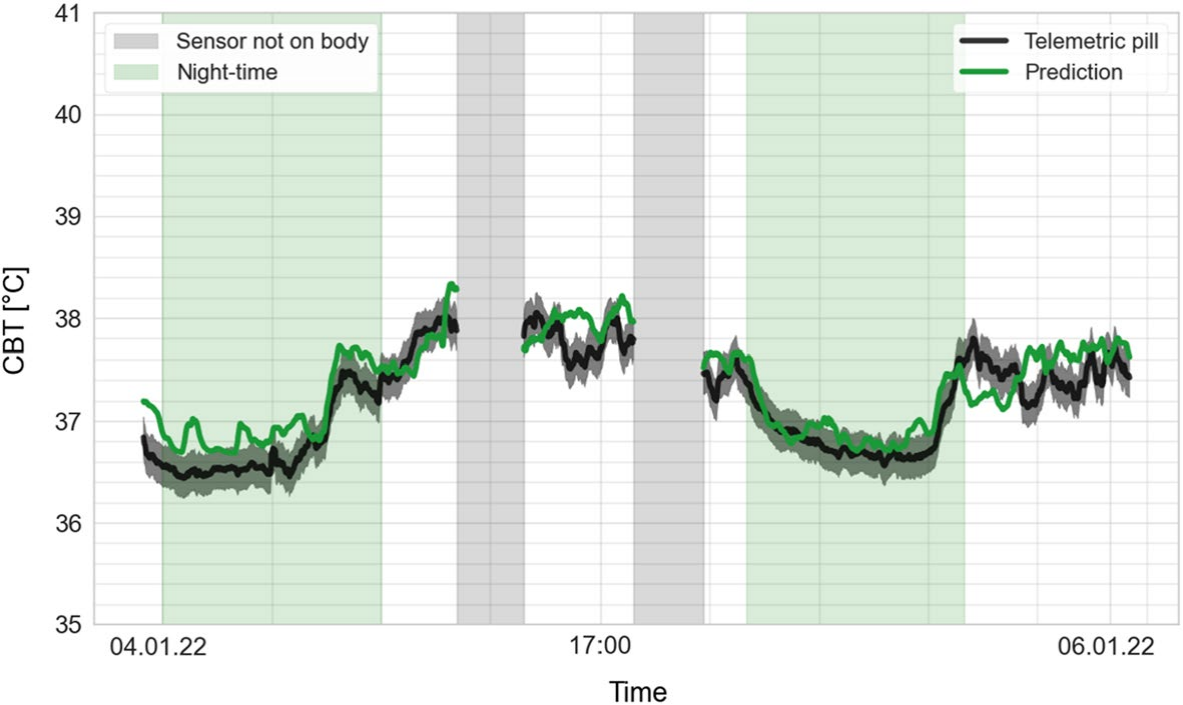
Predicted CBT and reference CBT over a participant’s recording time. The time the participant spent in bed is highlighted in green. Periods during which the participant did not wear the sensor are marked in light grey. The dark grey field around the black line represents the confidence interval for CBT as indicated by the fabricant of the ingested telemetric pill

In group two, the prediction mean absolute error (MAE) was 0.34 °C (standard deviation, SD 0.12). The Bland–Altman analysis shows good agreement between predicted CBT and reference CBT, with a bias of 0.11 °C (SD 0.23) (Fig. 2). The upper limit of agreement (LoA) was 0.93 °C and the lower LoA was − 0.67 °C. In Fig. 3, predicted CBT was plotted against reference CBT for each measurement. The correlation coefficient of the two measurement methods was *r* = 0.72 (SD 0.2). Overall, fever detection performance resulted in a false positive rate (FPR) of 14.9% and false negative rate (FNR) of 23.6%. Figure 4 shows a participant’s predicted and reference CBT over the entire observation time.

## Discussion

To the best of our knowledge, this is the first study to investigate continuous CBT monitoring and detection of elevated CBT with a wrist-worn device in free-living individuals. The technology had previously been shown to have high accuracy in detection of elevated CBT in the context of physical activity (integrated in the greenTEG® CORE® device) [29], as well as in a clinical context when placed on the chest [16]. There is no universally accepted definition of “fever” or for the upper limit of normal CBT [30]. For this study, we defined “CBT elevation” as elevation of CBT > 38.0 °C, a cut-off often used in clinical practice [1, 31]. Approximately 66% of the participants developed elevated CBT in the 24 h after the COVID-19 vaccination, which is slightly higher than previous observations [27, 28]. The precision of the wrist-worn CBT prediction system was comparable to that of other thermometers routinely used. A meta-analysis of 32 studies found similar mean bias and LoA compared to centrally measured CBT for tympanic membrane thermometers and axillary thermometers (− 0.08 °C [− 1.42 to 1.26 °C], and − 0.33 °C [− 0.94 to 0.27 °C], respectively) [7]. The false positive and false negative rates for the detection of elevated CBT (14.9% and 23.6%, respectively) are explained by the algorithm’s intrinsic tendency to average values and by the fact that it had previously been trained in normal CBT. Further training of the algorithm in the high CBT range is expected to further increase its accuracy.

Real-time CBT monitoring with a device allowing users to follow their daily routine is promising regarding several aspects. During the COVID-19 pandemic, RPM programs with VS and symptoms assessed several times a day and reported to health care professional in telemedicine consultations have shown to avoid short-term hospital admissions [23] and to allow early hospital discharge of infected patients [24, 32]. However, patients were provided with a large amount of technical equipment (blood pressure monitors, pulse oximeter, thermometer), and VS measurements as well as reporting of the results had to be performed manually. Gruwez et al. describe poor patient compliance after day 5 of the RPM program [25]. Furthermore, the repeated measurements and website or webcall-based reporting reduced the access to these programs for people with poor digital literacy. Thus, typically the elderly, who most likely would have benefited from monitoring programs, often were excluded from the RPM programs. The development of automated “all-in-one” devices that include a CBT sensor, may help to facilitate such initiatives by making VS assessment easier and improving compliance.

Global warming has increased attention about the limits of human adaptability to high environmental temperatures and has raised concern about vulnerable individuals’ health during the increasingly frequent heat waves [33–36]. Wearable CBT monitoring technology could be helpful to monitor persons at risk for heat-related illness during such episodes and thus to support the development of early detection programs.

Traditionally, CBT values have been interpreted dichotomously: patients either have a fever or are afebrile. Although used as indicator of disease since antiquity, “fever” has no universally accepted definition today [30]. The International Society of Physiological Studies (IUPS) defines fever as “a state of elevated core temperature” [37]. However, the meaning of “a state of elevated core temperature” is still debated in the clinical context [30]. Since the seminal works of Wunderlich in the nineteenth century [38], many efforts have been undertaken to define “normal” human body temperature, its deviations and the relationship between disease and temperature [1, 2, 31], and it has been demonstrated that body temperature is influenced by many factors, most importantly age and site of measurement [1]. Attempts have also been made to distinguish fever patterns and their significance, but none of these approaches was accurate enough to support clinical decisions [39]. However, recent evidence suggests that CBT pattern analysis can provide valuable clinical information, regardless of whether the patients meet fever threshold criteria, such as prediction of sepsis development [40], adverse events in immunocompromised hosts [41] and discrimination of bacterial vs. other cause of fever in patients hospitalized for suspected bacterial infection [42]. Continuous temperature monitoring is routinely used for perioperative temperature management [43, 44], and non-invasive zero-heat-flux and double sensor technologies have been studied for application in this context [9, 45, 46]. However, the placement of these devices’ probes on patients’ foreheads makes them unsuitable for use on general care wards. Ward monitor systems should use small, wireless and wearable sensors and be easy to use, giving hospitalized patients the freedom to move within their rooms and the health-care facility [47–49]. The technology tested in the present study has been shown to be a reliable alternative to tympanic membrane thermometers in hospitalized patients [16]. Evidence exists that automated monitoring of VS such as heart rate, oxygen saturation and blood pressure in patients hospitalized on the general care ward can improve patient outcome compared to intermittently measured VS [50–52]. Thus, there is potential utility of a small, wireless, and power-autonomous CBT sensor in the clinical context, especially if integrated into monitoring systems that also assess other VS.

We identified some limitations in our studies. All data collected included temperature patterns below 39 °C. The relatively mild CBT elevation induced by the second or third COVID-19 vaccine doses did not allow training and validation of the algorithm in higher CBT ranges. Second, the behavior and activity of most free-living individuals lacked variety. Future investigations should consider a variety of active daily life scenarios. An in-depth analysis of effects from environmental and contextual confounders is needed to better clarify the sensor’s ability of tracking fever patterns in outdoor free-living. Third, the relatively young age and high motivation of the study population recruited in a special study setting hinders extrapolation to the adherence to the device of other populations, such as elderly or ill patients.

## Conclusion

Continuous CBT monitoring and detection of elevated CBT with a wrist-worn sensor is reliable and accurate in free-living individuals. Continuous CBT monitoring has the potential to improve the surveillance of both patients included in remote monitoring programs as well as patients hospitalized on general care wards.

## Methods

### Participants and experimental protocol

The study was conducted over a 7-month period between September 2021 and March 2022 at the University Hospital Basel, Switzerland. Information about the study was provided to individuals presenting to the hospital’s employees’ COVID-19 vaccination center. Interested individuals were assessed for eligibility. Eligible participants were defined as those aged between 18 and 60 years and who received their second or third COVID-19 vaccination at the day of inclusion. Exclusion criteria were: inability to sign consent, inability to swallow pills, history of major gastro-intestinal surgery, ≤ 40 kg body weight, scheduled magnetic resonance imaging (MRI) examination in the period from the start of the measurements until seven days after ingestion of the telemetric pill, pregnancy, impairment or disability of the upper extremity likely to have a negative impact on the quality of measurements (e.g., wounds, active venous access, amputation, dialysis shunt, edema, axillary dissection, continuous long-term monitoring of blood pressure, tattoos), known allergy to plastic or latex, and language problems.

**Fig. 5:**
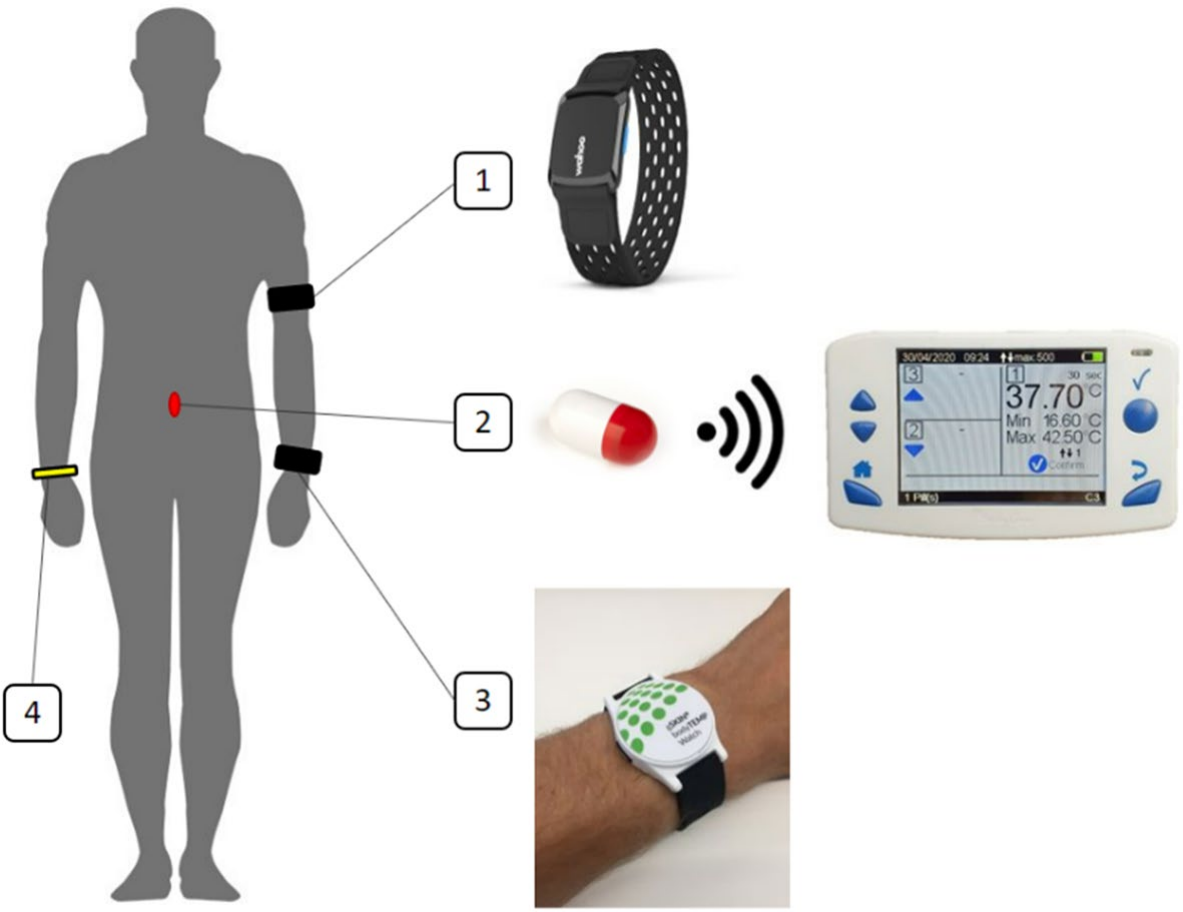
Schematic representation of the equipment provided to the participants: (1) optical heart-rate monitor on the left upper arm; (2) ingested telemetric pill and readout monitor; (3) wearable CBT sensor on the left wrist; (4) “no-MRI” warning bracelet

The participants were equipped with a prototype sensor containing the CALERA® CBT technology worn at the left wrist, and an optical heart rate monitor (Wahoo® Tickr fit®) placed on the participants’ left upper arm. All participants also ingested a telemetric temperature-sensing pill (eCelsius®, BodyCap, Caen, France) and received a corresponding readout monitor. A bracelet informing about the ingested telemetric pill and its limitation regarding MRI scans (no-MRI) was placed on the right wrist (Fig. 5).

After administration of the vaccine, participants were free to leave the vaccination center and to resume their usual activity. They were asked to record activities (e.g., meals, sleep, physical activity, showering, etc.) in a paper diary time sheet with hour-minute resolution. Continuous data were collected for a minimum of 12 h. The participants were instructed to perform a daily control of the data sent by the ingested telemetric pill with the corresponding readout monitor.

All procedures performed in this study were in accordance with the Declaration of Helsinki. The local ethics committee (Ethikkommission Nordwest- und Zentralschweiz, EKNZ) approved the study protocol and procedures (EKNZ 2021–00,690). All participants signed an informed consent before their participation in the study.

### CALERA sensor technology

The CALERA® technology (greenTEG®, Zurich, Switzerland) consists of a miniaturized heat flux sensor combined with skin temperature and heart rate sensors to continuously monitor CBT. A combination of physiological sensing, classical statistical modelling and embedded machine learning provides a CBT estimation at each one-minute sample. The heat flux signal is used to derive thermal resistance changes of the skin by compensating for skin temperature fluctuations caused by variations of the environmental conditions. CBT is estimated on-device and transmitted to a receiver by Bluetooth and ANT communication protocols. The small size of the heat flux sensor (2 × 2 mm) and its low power requirements facilitate the integration of the system into a wrist-worn device.

### Evaluation metrics

The CBT prediction performance was computed by comparing the model output with the reference CBT measured by the ingested telemetric pill. The following evaluation metrics were employed: (1) the bias, (2) the mean absolute error (MAE), and (3) the Pearson correlation coefficient:

1. Bias $$\frac{{\Sigma }_{i=1}^{n}{y}_{i} -{x}_{i}}{n}$$
2. MAE $$\frac{{\Sigma }_{i=1}^{n}{|y}_{i} -{x}_{i}|}{n}$$

where i indicates a one-minute sample, x_i is the CBT reference, y_i is the CBT prediction and n is the total number of samples; and

(3) Pearson correlation: ${{r}_{yx}=\frac{{\Sigma }_{i}{x}_{i}{y}_{i}-n\overline{x}\overline{y}}{\sqrt{{\Sigma }_{i}{{x}^{2}}_{i}-n{\overline{x}}^{2}}\sqrt{{\Sigma }_{i}{{y}^{2}}_{i}-n{\overline{y}}^{2}}}}$where $\overline{x}$ is the CBT reference sample mean, $\overline{y}$ is the CBT prediction sample mean.

All metrics were calculated sample-wise (i.e., on a minute-to-minute basis) on individual participant data. A correlation scatter plot was used to visualize the sample-wise correlation between prediction and ground truth, and a Bland–Altman plot was used to identify any patterns or biases in the residuals across the range of ground truth and to calculate limits of agreement (LoA). The bias was calculated by averaging all the one-minute errors, where ground truth was subtracted from the predicted signal. A positive mean bias indicates that the prediction overestimates the ground truth and a negative mean bias indicates that the prediction underestimates the ground truth.

Considering an elevated CBT event any sample higher than 38.0 °C, we also calculated elevated CBT detection performance by employing false positive rate (FPR) and false negative rate (FNR), as follows:

FPR = $$\frac{\mathrm{FP}}{\mathrm{FP }+\mathrm{ TN}}$$

and

FNR = $1- \frac{\mathrm{TP}}{\mathrm{TP }+\mathrm{ FN}}$,where FP is the number of samples wrongly categorized as elevated CBT events, TN is the number of samples correctly categorized as non-elevated CBT state, TP is the number of samples correctly categorized as elevated CBT events, and FN is the number of samples wrongly categorized as non-elevated CBT state.

## Data Availability

Restrictions apply to the availability of these data, which were used under license for this study. Data are available SE, with the permission of greenTEG AG.

## Abbreviations

CBT: Core body temperature
COVID-19: Coronavirus disease 2019
RPM: Remote patient monitoring
SARS-CoV-2: Severe acute respiratory syndrome corona virus 2
VS: Vital signs
